# Unraveling the regulation of mTORC2 using logical modeling

**DOI:** 10.1186/s12964-016-0159-5

**Published:** 2017-01-19

**Authors:** Kirsten Thobe, Christine Sers, Heike Siebert

**Affiliations:** 1 Group for Discrete Biomathematics, Department for Mathematics and Computer Science, Freie Universitaet Berlin, Arnimallee 7, Berlin, 14195 Germany; 2 International Research School for Scientific Computing and Computational Biology, Max-Plank Institute for Molecular Genetics, Berlin, Germany; 3 Laboratory of Molecular Tumor Pathology, Institute of Pathology, Charité Universitätsmedizin Berlin, Berlin, Germany

**Keywords:** Logical modeling, mTORC2 regulation, Cancer signaling

## Abstract

**Background:**

The mammalian target of rapamycin (mTOR) is a regulator of cell proliferation, cell growth and apoptosis working through two distinct complexes: mTORC1 and mTORC2. Although much is known about the activation and inactivation of mTORC1, the processes controlling mTORC2 remain poorly characterized. Experimental and modeling studies have attempted to explain the regulation of mTORC2 but have yielded several conflicting hypotheses. More specifically, the Phosphoinositide 3-kinase (PI3K) pathway was shown to be involved in this process, but the identity of the kinase interacting with and regulating mTORC2 remains to be determined (Cybulski and Hall, Trends Biochem Sci 34:620-7, 2009).

**Method:**

We performed a literature search and identified 5 published hypotheses describing mTORC2 regulation. Based on these hypotheses, we built logical models, not only for each single hypothesis but also for all combinations and possible mechanisms among them. Based on data provided by the original studies, a systematic analysis of all models was performed.

**Results:**

We were able to find models that account for experimental observations from every original study, but do not require all 5 hypotheses to be implemented. Surprisingly, all hypotheses were in agreement with all tested data gathered from the different studies and PI3K was identified as an essential regulator of mTORC2.

**Conclusion:**

The results and additional data suggest that more than one regulator is necessary to explain the behavior of mTORC2. Finally, this study proposes a new experiment to validate mTORC1 as second essential regulator.

## Background

The mammalian target of rapamycin (mTOR) is a highly conserved kinase across species, from yeast to humans, playing a central role in coordinating cell growth, metabolism and survival of the cell [1]. In the cell, mTOR acts as a signal integrator through two distinct complexes, mTORC1 and mTORC2, each phosphorylating distinct sets of substrates upon stimulation by growth factors, nutrients, hormones, stress, and other stimuli [2]. Dysregulation in these processes was found to be present in many cancer types, therefore understanding the structure and dynamics of mTOR regulation is of high interest [3, 4]. Although mTORC1 was the main focus of most studies so far, recent studies found mTORC2 playing an important role in cancer development, e.g. in HER2/PIK3CA-hyperactive breast cancer [5]. The development of novel mTOR kinase inhibitors has already yielded interesting findings on mTORC1 and mTORC2, but in order to successfully apply these drugs in combined therapy, a detailed understanding of the signaling processes is essential and not yet achieved [6, 7].

**Fig. 1:**
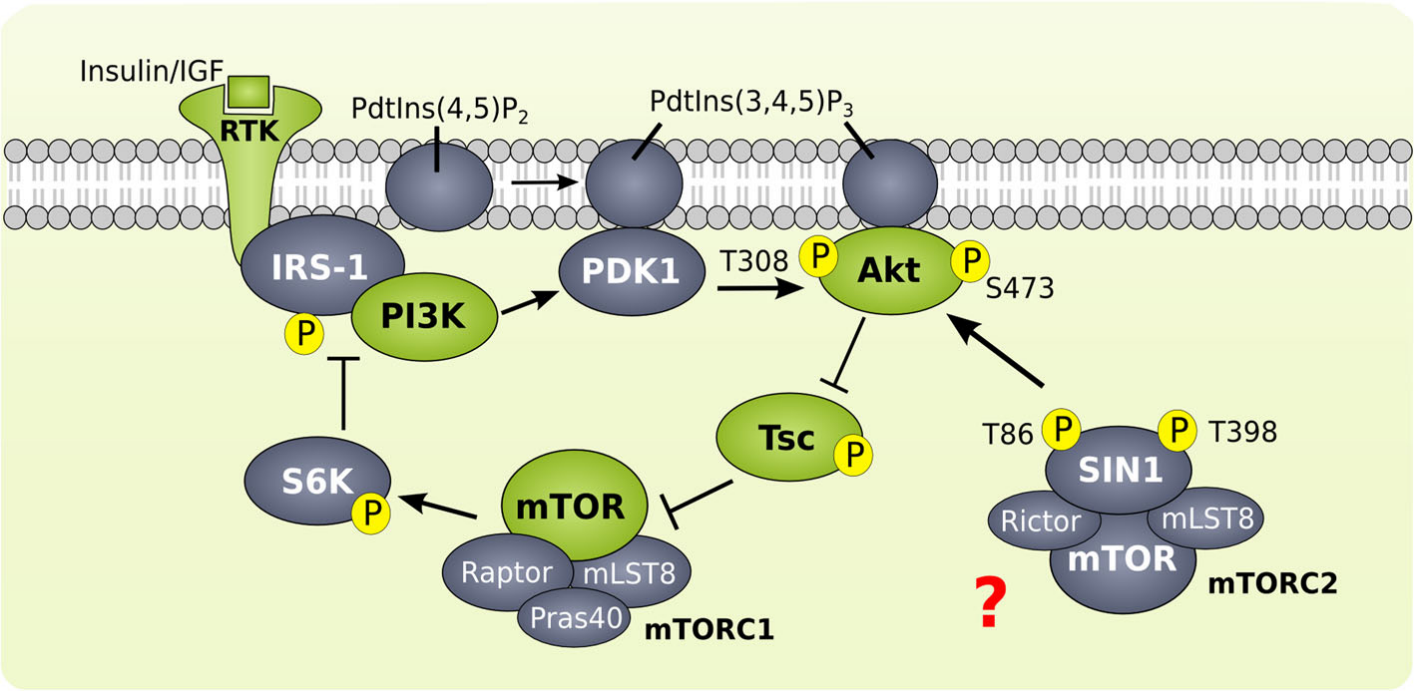
Scheme of PI3K pathway with candidate regulators of mTORC2 colored in *green*. Insulin and growth-factors activate RTK signaling through PI3K and the well-known regulation of mTORC1 with negative feedback on IRS-1. The regulation of mTORC2 is unclear

Besides the catalytic mTOR subunit, both complexes contain mLST8, while Rictor and SIN1 are specific for mTORC2 and Raptor and Pras40 are specific for mTORC1. Upon stimulation, receptor tyrosin kinases (RTK) activate mTORC1 mainly via PI3K-mediated phosphorylation of Akt (see Fig. 1). In detail, the activated receptor binds and activates IRS which recruits PI3K to the plasma membrane. PI3K activates PDK1 binding *PdtIns*(3,4,5)*P*_3_ (PIP_3), which recruits the multi-functional kinase Akt to the plasma membrane and phosphorylates it at T308. This phosphorylation is sufficient to activate Akt which in turn inhibits one of its targets, the tuberous sclerosis complex 1/2 (Tsc) [8]. This process activates the G protein Rheb, which then activates mTORC1 [9]. One main target of mTORC1 is p70S6K, which is able to phosphorylate IRS. Thereby the binding of IRS to PI3K is disrupted and PI3K becomes inactive, thus creating a negative feedback [10]. Upon stimulation with growth factors, mTORC2 activates AGC kinases (protein kinase A, G, and C), in particular it phosphorylates Akt at S473 [11]. Moreover, mTORC2 was reported to phosphorylate Akt at T450 at the mitochondrial membrane as a protein stabilizing post-translation modification. However, this process is growth-factor independent and not considered here.

### Conflicting studies on mTORC2 regulation

In contrast to mTORC1, the processes that control mTORC2 are uncertain [12]. There are multiple studies investigating the influence of various kinases from the PI3K pathway on mTORC2 or components of its complex. Each of these studies is used as one hypothesis in our investigations: feedback independent regulation via RTK [13], activation by PI3K [14], positive feedback from Akt on mTORC2 [15], Tsc dependent regulation [16], and inhibition by mTORC1 [17].

**Hypothesis 1: Feedback independent activation** A feedback independent activation of mTORC2 was proposed by Dalle Pezze et al. [13], where they presented a data-driven ODE modeling approach investigating three different models: one model having Tsc as activator, a second model with only PI3K as activator and a third model where mTORC2 is regulated by an unknown kinase, which is independent from the negative feedback on PI3K [13]. Here, the authors used experimental design based on simulations of the models to find perturbation experiments that are able to distinguish between the different hypotheses. Thereby, the group was able to extract their final model as a feedback independent activation of mTORC2.

**Hypothesis 2: Direct activation by PI3K** An activation of mTORC2 by PI3K was proposed by two different groups. Gan et al. [14] claimed that it is known that PI3K via PIP_3 has two effects on Akt. First, it recruits the kinase to the plasma membrane and phosphorylates the protein at T308. Secondly, it regulates the S473 phosphorylation of Akt via mTORC2, but whether or not it directly interacts with the complex is unknown [14]. Therefore, the authors created an Akt mutant which is constantly bound to the plasma membrane and thereby dissecting the recruiting effect from PIP_3 from its potential activation of mTORC2. Although they were able to show that the regulation via the Akt mutant is still sensitive to PI3K inhibitors, the exact mechanism could not be clarified [14].

In a recent work by Liu et al. (2015) a regulation of mTORC2 by PI3K was claimed, where they observed molecular interactions between SIN1, Akt and PIP_3 [18]. Liu et al. (2015) suggested that SIN1 might act as gate-keeper in mTORC2, therefore they investigated its mechanistic interaction with mTOR. As a result, the experiments showed that an interaction of SIN1-PH domain with the kinase domain of mTOR leads to a suppressed mTOR activity [18]. Since PH domains are characterized by their ability to bind *PdtInsP*_*n*_ *s*, Liu et al. (2015) tested binding properties of different *PdtInsP*_*n*_ *s* to SIN1-PH. They showed that PIP_3 binds to the SIN1-PH domain. Moreover, PIP_3 and SIN1 were shown to compete for binding with the kinetic domain of mTOR. Therefore Liu et al. (2015) claim that SIN1 binds mTORC2 blocking its activity and PIP3 then binds SIN1 to release the inhibition on mTORC2, then Akt can bind to be phosphorylated.

**Hypothesis 3: Akt directly activates mTORC2 causing a positive feedback** Another member of the PI3K pathway, Akt, was proposed to regulate mTORC2 by two studies from the James lab [15, 19]. First, Humphrey et al. presented a quantitative analysis of the insulin signaling network in adipocytes using mass spectrometry-based proteomics [19]. In particular, they suggested that SIN1 phosphorylation at T86 is insulin sensitive and that this regulation acts through Akt, due to its timing and Akt inhibitor response. Moreover, a recent paper from the same lab by Yang et al. showed the same effect on a molecular level in various cell types [15]. Here, they examined SIN1 phosphorylation at T86 upon Akt, mTORC1 and S6K inhibition, showing a reduced phosphorylation level only for Akt inhibition but not mTORC1 or S6K inhibition. They conclude that the activation of mTORC2 follows activation of Akt by T308 phosphorylation, then Akt phosphorylates SIN1 activating mTORC2, which itself then phosphorylates Akt at S473 for its full activation [15].

**Hypothesis 4: Activation by Tsc2** Huang et al. [16] found that Tsc2, a component of Tsc, is required for mTORC2 activity by performing experiments with Tsc knock-out Mouse Embryonic Fibroblast (MEFs). For various stimuli they showed that in these cells the phosphorylation of Akt at S473 is lacking, but can be recovered adding a vector that expresses human Tsc2 [16]. Due to the negative feedback of mTORC1 on PI3K, a decreased activity of mTORC2 in Tsc2 knock-out cells can also result from constantly active mTORC1. In the paper, Huang et al. argue that the effect of the Tsc2 knock-out can be separated from the feedback by looking at experiments with mTORC1 inhibition.

**Hypothesis 5: Integrity of mTORC2 is regulated by mTORC1 via SIN1 phosphorylation** In direct contradiction with the findings of Humphrey and Yang et al., Liu et al. (2013) claimed in an earlier paper that S6K or Akt phosphorylates SIN1 not only at T86 but also at T398 and thereby causes a dissociation of the mTORC2 complex resulting in its inhibition [17]. In this paper, HeLa cells and MEF cells were stimulated with either insulin or EGF and treated with various inhibitors mostly rapamycin but also S6K and Akt inhibitors. Moreover, SIN1 mutants with T96A and T398A genotype were used to mimic permanently non-phosphorylated SIN1 variants as well as knock outs.

### Modeling of uncertain systems

In order to clarify the regulation of mTORC2, we use mathematical modeling to systematically analyze the proposed hypotheses from the literature. When modeling an uncertain system, one can either build a model based on assumptions or build every possible model that arises from the uncertainty to compare their performance. However, depending on the modeling formalism building every possible model can be computationally challenging, e.g. finding parameters for one ODE model is already a hard problem usually also rife with uncertainty. Here, we use a logical modeling workflow [20–22] to create and analyze possible topologies and mechanisms of biological systems. This formalism is able to capture qualitative effects of the interactions by analyzing basic behaviors, which was shown to deliver valuable results for signaling processes [23–25]. Other logical modeling approaches that incorporate uncertainty are available, e.g. CellNetOpt or an Answer Set Programing based approach by Videla et al. being similar to our tool [26, 27]. These tools differ in several aspects from our approach. In particular, both methods train models according to optimality criteria rather then considering the full set of consistent models, or focus on steady state responses.

For our approach, available information about the biological system is collected from literature and represented in a graph: components as nodes and interactions as edges. Then, the quality of this information is evaluated, where highly certain and textbook knowledge is considered to be essential and uncertain information is considered to be optional. For our model this means that interactions arising from essential information are identical in every model whereas optional interactions can be present or absent. In the graph, this evaluation is given by edge labels, such as for an activating interaction the essential label is + and for an optional edge we define it as not inhibiting ¬−, which means the edge is either absent in a model or it is activating.

The model is then formed by defining logical rules for every component of the graph describing the regulatory wiring of the incoming edges using logical AND or OR connections. In case a graph contains an optional edge, multiple models are build for either not containing that edge or containing it and then building every possible logical rule with it. Thus, building the set of all models, called model pool, is a combinatorial problem that grows with the number of optional edges. By simulating qualitative behavior of a model, we can compare the dynamics of each model to available data in order to find those models that are consistent with the data while eliminating others from the pool. Finally, the consistent models can be analyzed using specialized software in order to eliminate uncertainties and identify trends for further studies (for more details see “Methods” section).

Here, we identified 5 hypotheses for the regulation of mTORC2, which are translated to uncertain edges on mTORC2 and give rise to the model pool. Testing this pool for data from the original studies posing these hypotheses markedly reduced the size of the model pool. By analyzing this pool, we found that none of the hypotheses contradict the data from other studies, even though they intended to support a different hypothesis. Also, we found that some interactions are redundant and therefore not necessary for the model. Thus, we identified models with less than five edges that are valid for data sets from all original studies. Since biology is expected to be sparse, we sought the simplest mechanism by looking at minimal models. Such a minimal model is having PI3K as the only regulator of mTORC2, moreover we found this regulation to be essential.

The formalism allows for easy implementation of in silico experiments, which in turn can be exploited for wetlab experimental design. Formal analysis indicates that the feedback is a crucial factor for obtaining informative data. To illustrate this point, we propose and implement in silico a simple experiment that blocks the feedback. Exploiting the resulting data for the pool analysis shows that this experiment could validate mTORC1 as a second essential regulator additionally to PI3K, which is supported by recent publications.

## Results

### Model building from literature

For building a model of the mTORC2 regulation by signaling processes, we only included studies investigating direct interactions with the complex, excluding metabolic effects. We reduced the biological system to those components that are measured or perturbed in the studies we examined. The interactions between these components and their labels are also deduced from literature, where interactions that are widely accepted to be common knowledge are set to mandatory and uncertain interactions as optional (for more details on the formalism see “Methods” section). Here, the regulations within the PI3K pathway and the negative feedback are assumed to be known. However, the regulation of mTORC2 is unclear, thus all candidate interactions from RTK, PI3K, Akt, Tsc and mTORC1 are set as optional.

The regulations of the components are defined as functions according to the edge labels. For components that only have one regulator, the function can be directly derived from the edge label. For PI3K and Akt, the logical connection between the regulators needs to be deduced from biological knowledge. PI3K is activated by RTK and inhibited by IRS, where IRS binds and thereby blocks PI3K from interaction with other components including RTK. Thus, the logical connection is AND, since PI3K can only be active if RTK is active and IRS is not. The interactions from PI3K and mTORC2 on AKT are connected with a logical OR, because both are able to activate the component independently [8].

**Fig. 2:**
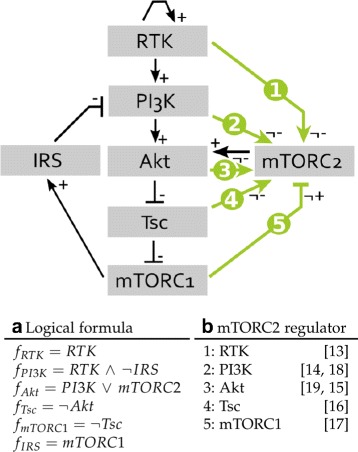
Interaction graph and overview of hypothesis for mTORC2 regulation. *Black lines* indicate edges that are mandatory and *green* lines have edge labels allowing for uncertainty annotated with their respective edge label. **a** List of logical functions for components with known regulation, where the notation signifies a logical AND as ∧, OR as ∨ and negation as ¬. **b** List of candidate regulators for mTORC2 in the literature

The interaction graph of the model is shown in Fig. 2, with the logical functions on the left side and a list of the regulators with references on the right. For the regulation of mTORC2 various hypotheses were published, which can be summarized as 5 candidate regulations:

1. RTK indirectly regulates mTORC2, thus the regulation is insensitive to the negative feedback of mTORC1 on PI3K [13]. For our model, we included an activating edge from RTK to mTORC2 with the label not inhibiting.
2. PI3K directly activates mTORC2 [14, 18], thus we include an activating edge from PI3K to mTORC2 labeled as not inhibiting in our model as Hypothesis 2.
3. Akt activates mTORC2 by phosphorylation of SIN1 at T86 [15, 19]. For our model, Hypothesis 3 is a not inhibiting edge from Akt to mTORC2.
4. Tsc is required for mTORC2 activity shown in Tsc2 knock-out cells [16], therefore we included this observation as Hypothesis 4 as a not inhibiting edge from Tsc to mTORC2.
5. A phosphorylation of SIN1 by S6K causes a disintegration of the complex [17], thus we included an inhibiting edge from mTORC1 on mTORC2 with the label not activating into our model.

In Fig. 2 these regulations are marked as green lines, meaning that we do not know whether these connections are functional. Thus, the logical function for mTORC2 is uncertain and we want to explore all possible models using the edge labels and known logical rules for the other components as constraints. For building the model pool, every topology of possible combinations of the 5 candidate edges is created, resulting in 32 topologies. Then, for every topology all truth tables, representing a logical function, are generated and subsequently selected for the pool if they agree with the constraints (for more detail see [20]). This process is computationally challenging, e.g. for a component with *n* optional incoming edges the upper bound of possible truth tables is $2^{2^{n}}$. Using the software Tremppi [21], a model pool of 7581 models is determined.

### Discretization of experiments from literature studies shows redundancy in data

Subsequently, data from the original studies were used to filter the model pool for those models that are in agreement with the experimental data. For this aim, we need to discretize this data to match the logical formalism and encode it to make it accessible for our software. The discretization method depends on the data type, e.g. quantitative values or western blot images (for detailed description see the “Methods” section). Here, we discretized data from each study which was included as a hypothesis for this investigation. Since the original studies have different levels of detail and used different methods to prove their hypothesis, we can only include a subset of the performed experiments here (see Discussion for more details). However, even though the papers claim different results, we found that many performed the same or similar experiments from a qualitative perspective.

For example, the time series measurements upon insulin stimulation were done by five out of seven studies. The resulting discretized sequences were partially redundant with data sets of other experiments (see Table 1). Similarly, an experiment with mTORC1 inhibition and insulin stimulation was done by four groups, either using rapamycin or shRNA against Raptor. After discretizing the data, all studies observed active PI3K and mTORC2 measured by Akt phosphorylation. The effect of PI3K inhibition on mTORC2 activation was studied by four groups, where inhibitors like Wortmannin or LY294002 were used to directly block PI3K or the activating connection to Akt was impeded by inhibiting PDK1. These experiments consistently led to inactive mTORC1 and mTORC2 across all studies. Three studies investigated the activity of mTORC2 upon insulin stimulation in Tsc knock out/down MEF cells with equivalent results.

**Table 1:** Redundancy in experiments across different studies

	PI3K inhibition	mTORC1 inhibition	Tsc knock out	Insulin stimulation
Dalle Pezze et al.	Fig. 8a	Fig. 7a	Fig. 6a, b	Fig. 4a, b
Gan et al.	Fig. 2a			
Liu et al. (2015)	Fig. 3d			Fig. 2d
Humphrey et al.				Fig. 6b
Yang et al.	Fig. 4c	Fig. 4a, b		Fig. 4b
Huang et al.		Fig. 3a	Fig. 1a	Fig. 3
Liu et al. (2013)		Fig. 1a	Fig. S4j	

The columns show types of experiments that were done in various studies yielding in matching qualitative behavior after discretization (data not shown)

For our study, we included the most comprehensive data set for our analysis and excluded redundant information.These comparisons reduced the number of data sets to be tested to five different experiments shown in Table 2. Note that this observation indicates a certain reliability of the data, since even though the experiments were performed by different groups with different aims and setups, their qualitative interpretation is comparable.

**Table 2:** Data processing for logical analysis by discretization and formal encoding as CTL formula

	

The tables show measured components, time points in minutes and readout. For the CTL formulas the settings are given, which is the measurements, the initial state and fixed components. If no measurement at time point 0 is available, the set up of the experiment is used, e.g. stimulation of the receptor. The fixed components encode a knock down/out in that component. Additionally, the option Delta=0 encodes a steady-state (more details see “Methods” section). **T_4B** Time series data of selected components from Fig. 4b in [13]. The table shows measurements that were discretized by mean value. CTL formula uses time point 0 as initial state and further data points as sequence. **T_7A** Perturbation experiment with knock down of mTORC1 component Raptor leads to sustained Akt activity, encoded as fixpoint in the CTL formula with fixed mTORC1 (Fig. 7 in [13]). **T_8A** PI3K inhibition by Wortmannin causes complete inhibition of all pathway components including Akt and mTORC1 target p70-S6K (Fig. 8 in [13]). The Data in encoded as a fixpoint with fixed PI3K. **M_1A** Data from Huang et al., where Tsc2-/- cells show inactive mTORC1 and mTORC2, encoded as fixpoint with fixed Tsc (Fig. 1 in [16]). **M_3BC** and **M_3BC2** Combined data sets from two experiments for showing the independence of Tsc effect on mTORC2 and negative feedback (Fig. 3 in [16]), encoded as fixpoint with Tsc and IRS fixed

In order to compare the data with the model dynamics efficiently, the discretized data needs to be encoded in temporal logics, here we used Computation Tree Logic (CTL). For this encoding, each measurement is defined as a state of the system that needs to be reached from the previous measurement. Additionally, the temporal interpretation of a state needs to be defined, meaning that the state can be an initial state, a transient state or a steady state (see “Methods” section). In the following, the discretized and encoded experimental data included in this study is described in brief.

We used time-course measurements as well as knock down experiments from the study of Dalle Pezze et al. [13]. In detail, time series measurements of insulin stimulated HeLa cells for various proteins were done (see Fig. 4 in [13]). Here, we included data of the following components in our model: Akt-pT, Akt-pS, IRS-pS, and S6K-pT (see Table 2 **T_4B**). The data was discretized by mean value, then assigned to its designated readout. Here, RTK was added to the data set as a measured component to encode the stimulation over time. Finally, the sequence is encoded as CTL shown in Table 2 **T_4B**.

We also included data from the perturbation experiments from Dalle Pezze et al., where mTORC1 was inhibited by shRNA against Raptor in HeLa cells and the phosphorylation levels of Akt were measured 45 and 100 minutes after insulin stimulation (see Fig. 7 in [13]). The corresponding CTL formula in Table 2 **T_7A** contains RTK as active in the initial state due to insulin stimulation and the knock down of Raptor is encoded as setting the logical equation of mTORC1 to 0. We call this a fixed component. Moreover, we assume the signaling process to be in steady state, since there is no change even after 180 minutes observable.

The effect of PI3K inhibition on mTORC2 activation was studied by treating HeLa cells with different concentrations of the inhibitor Wortmannin, which directly blocks PI3K (see Fig. 8 in [13]). After stimulating the cells with insulin, inactive mTORC1 and mTORC2 was measured after 30 and 50 minutes, where the effect intensified with increasing concentration. The resulting CTL formula T_8A is shown in Table 1, where PI3K is fixed to zero due to the inhibition and the dynamics are assumed to be a fixpoint, since the behavior was stable over both time points.

Huang et al. used *Tsc*2^−/−^ Mef cells and treated them with various stimuli for 30 minutes to measured Akt-pS as well as S6K-pT (see Fig. 1a in [16]). To encode the knock out, Tsc is fixed to zero and the stimulation is encoded as active RTK. These experiments lead to active mTORC1 but inactive mTORC2 after e.g. insulin stimulation, resulting in the CTL formula **M_1A** (see Table 2). The authors expected this behavior to be stable over time, therefore we encoded this measurement as a fixpoint.

Also, Huang et al. investigated the influence of the negative feedback on the signaling process. In Fig. 3b and c in [16], *Tsc*2^−/−^ Mef cells were treated with insulin for 15 minutes and Akt-pS, IRS and its binding to PI3K was measured. In the experiment, the phosphorylation of IRS by mTORC1 was measured showing a hyperphosphorylation due to the knock-out in the mTORC1 inhibitor Tsc. In this hyperphosphorylated state of IRS the binding with RTK and PI3K disintegrates and PI3K becomes inactive, thus IRS is fixed to 1 in the CTL formula **M_3BC**. Additionally, they claimed that the impaired mTORC2 activity in *Tsc*2^−/−^ Mef cells was not caused by a constantly activated feedback through mTORC1 on PI3K [16]. They argued that the mTORC2 activity should be rescued upon mTORC1 inhibition causing a deactivation of the feedback, if PI3K would be the only activator. To show this, mTORC1 was knocked down using siRNA against Raptor and the cells were stimulated with insulin for 15 minutes. In Fig. 3b and c in [16], the binding of IRS to PI3K was restored, but the mTORC2 complex did not show any kinetic activity. For the CTL formula **M_3BC2**, Tsc and mTORC1 are fixed to zero. The data sets **M_3BC/2** are examined separated from the other experiments, because 15 min measurements are usually not sufficient to assume a fixpoint. However, we are especially interested in the effect of the feedback on the dynamics of the models.

Additionally to the experimental data, we assume that without any stimulus the signaling system should reach an inactive steady state. This steady state represents the quiescence state of the biological system that is supposed to be fulfilled for the highly regulated growth-factor signaling in healthy tissue. Formally encoded, this means **Triv_FP**: EF(mTORC2=0 & Delta=0), Initial State: RTK=0.

**Filtering for data reduced size of model pool** Based on the data, we were able to fully determine the regulation for every component in the model only the regulation of mTORC2 remains to be elucidated (Fig. 2). Combining all possible logical functions from 5 optional edges under the given constraints gives rise to 7581 models, the set of which we call initial pool. In the next step, this pool is filtered by applying CTL formulas derived from the data in Table 2 as restrictions on the model pool using model checking. Thereby, the trajectories of each model are matched with the sequences defined by the CTL formulas and the model is rejected in case of a mismatch.

As a result, each CTL formula reduces the initial pool to subpools of various sizes (see Table 3). Finally, the intersection of these subpools creates different reduced pools, which contains only those models that are valid for all CTLs. The main reduced pool we obtained is called Red.pool having 944 models that are true for all data sets excluding **M_3BC** and **M_3BC2**. If we now apply additional CTL formulas to the Red.pool, the intersection with **M_3BC** does not result in a further reduction in the pool size meaning that all models in the Red.pool are valid for this data set. However, the opposite is true for **M_3BC2**, where no model from the Red.pool agrees with this formula. We will discuss this point further in the following section.

**Table 3:** Applying CTL formulas to the pool reduced its size markedly

CTL:	/	Triv_Fp	T_4B	T_7A	T_8A	M_1A	M_3BC	M_3BC2	ExpD1	ExpD2
# models:	7581	5573	5202	7413	2008	7413	5573	168	2008	5573
:		Red.pool: 944					944	0	310	634

Red.pool is the intersection of all data sets except M_3BC and M_3BC2. M_3BC shows no further reduction on the Red.pool, whereas M_3BC2 has no shared models with the Red.pool. On the right, the experimental design formulas are shown, which both show a further reduction on the selected pool

### Model pool analysis

In oder to gain information about the influence of the applied data on the pool, we need to analyze the pools. Although filtering for the data reduced their size markedly, they are still too large to analyze them by hand. Therefore, we employ a statistical approach first, following up with an exact analysis.

**Strong influence of PI3K regulation shown in statistical analysis** In a first step, we evaluated both the reduced and the initial pool statistically using the software Tremppi [28], which is an efficient model checker tool developed for these applications. In the statistical analysis, we want to identify edges that are enriched or under-represented in the filtered pool in comparison to the initial pool, since this difference is strongly implied by the data [22]. For this purpose, two measures are estimated: (i) frequency of an edge and (ii) its impact. For (i), the frequency of appearance of an edge across the pool is counted and divided by the pool size. The higher the frequency, the more models require the considered edge to be present. Then in (ii) the impact of the regulator on its target is calculated as correlation coefficient in the range [-1,1] between source and target component in every model, where -1 means fully inhibiting, 1 is fully activating and 0 means no influence. The impact is the averaged correlation across the pool. A high impact indicates that the regulator across all models in the pool has a strong influence on the behavior of the target, since their states are highly correlated.

**Fig. 3:**
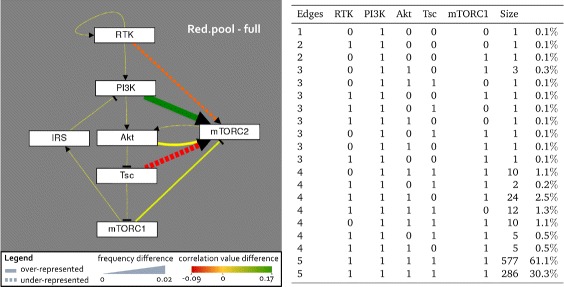
PI3K regulation on mTORC2 is present in every filtered model, but not in original pool. Statistical analysis of the reduced pool and initial pool for frequency F and impact I (correlation of components) was created with Tremppi and the graph shows the difference (reduced - full) for the Red.pool. PI3K regulation of mTORC2 is overrepresented in the reduced pool in both frequency and impact compared to the initial pool. The regulation by RTK and Tsc is less frequent in the filtered pool than in the full shown by *dashed lines*, *yellow dotted lines* show identical frequency and impact in both pools. The table shows the classification of all 994 models in the Red.pool according to the following features: Edges in the model, the data sets, and active hypotheses. Size gives the number of models in the class and the percentage of this class in the pool

For our analysis, we evaluated the initial pool and the Red.pool, then calculated the differences between them by subtraction. In Tremppi, these differences can be visualized as a graph shown in Fig. 3. Here a graph illustrates the difference between the Red.pool and the initial pool, where an increase in frequency and impact for the regulation of mTORC2 by PI3K can be observed (Fig. 3). Moreover, the Red.pool contains less models with a regulation by RTK and Tsc than the initial pool and the impact of these edges is reduced. Note that the frequency and correlation differences are very small, since for frequency the maximum value is 0.02 in possible range of [0,1] and the correlation ranges between -0.09 and 0.17 with a possible range of [-2,2]. This means that the difference in frequency and impact between the pools is small, which can be explained as an artifact of the modeling process (more information in Discussion section). In order to resolve these results further, we examine the composition of the pools explicitly.

**Minimal model corresponds to Hypothesis 2** Despite the fact that the statistical evaluation is able to give us important information about the changes in the pool composition, it does not give information about explicit models. Thus, we used a second model checking tool called TomClass [29], which groups the models into classes according to defined features. Since we are interested in the topology of models and their dynamical behavior, we defined the features as: number of edges, validation for the CTLs, and present hypotheses. Then all models that are equal with respect to these features are grouped in one class, which in our setup means, models in one class share the same topology and behavior towards the checked CTLs, and only differ in their logical equation.

Figure 3 shows all 994 models in the Red.pool containing models with less than 5 edges. However, adding up the size of classes with 5 edges, it becomes clear that more than 90% of the models contain 5 edges as is expected, since the more regulators are available the easier the model can be fitted to the data. Moreover, we can say that all hypotheses are in agreement with the data, since every hypothesis is present in at least one model, even when only considering the models with less than 5 edges. In detail, 3 edges are necessary for all hypotheses to be present, for 2 edges models with pairwise combinations of mTORC1, RTK and PI3K are observed and only PI3K appears as possible single regulator. Thus, the minimal model, meaning the lowest numbers of mTORC2 regulators, corresponds to Hypothesis 2. Surprisingly, this edge is present in every model in the pool and therefore seems to be essential for the model dynamics to match the data. Thus, although all hypotheses are able to match the data, not all of them are necessary to be present.

**Analysis of additional data set causes conflict** We were especially interested in a data set by Huang et al., since they claimed to show an effect on mTORC2 that can be separated from the feedback affecting IRS and PI3K [16]. Two CTL formulas, **M_3BC** and **M_3BC2**, were extracted from this data set and applied both first as transient measurements. As a result, both formulas were in agreement with every model in the pool, since reaching one state is too easy for these very similar models in the pool (data not shown). Although the measurement time point was 15 minutes and therefore usually does not qualify for a steady state assumption, we tested the data as hypothetical fixpoints. Then, **M_3BC** was met by many models in the pool and the intersection with the Red.pool did not result in a further reduction of the pool (see Table 3).

However, the second formula **M_3BC2** led to a strong reduction in the pool size with only 169 out of 7581 being in agreement. When calculating the intersection with the Red.pool, the result is an empty set, caused by a direct conflict with **T_7A**. Therefore, our model does not support the conclusions drawn in the original paper (we will resolve this in more depth in the Discussion).

### Experimental design

The idea of the experiment in Huang et al. to disrupt the feedback for dissecting the processes in the cascade and their effect on mTORC2 led us to propose a new experiment. For this experiment, we want to eliminate the negative feedback, e.g. by mutating the target phosphorylation side in IRS such that IRS maintains its function as mediator of the signal from RTK to PI3K, but S6K cannot phosphorylate and inhibit PI3K. In such a system, a standard experiment would be to stimulate the receptor with insulin and measure the mTORC2 activity by AktpS levels.

From a modeling perspective, steady state measurements more effectively restrict the pool than transient measurements, therefore AktpS should be measured at multiple time points to ensure stability. The possible outcome of this experiment would be active or inactive mTORC2. To test this behavior on the Red.pool, we formulated these scenarios as CTL formula:

- **ExpD1**: EF(mTORC2=0 & Delta=0),
Initial State: RTK=1, Fix: IRS=0,
- **ExpD2**: EF(mTORC2=1 & Delta=0),
Initial State: RTK=1, Fix: IRS=0.

**Experiments split the pool for mTORC2 behavior** The CTL formulas split the initial pool as well as the Red.pool in two groups, showing that every model reaches a fixpoint (Table 3). In both cases, the pool for **ExpD1** is roughly half the size of **ExpD2** with 310 to 634 models for the intersection with the Red.pool, called Red.ExpD1 and Red.ExpD2 respectively.

**Fig. 4:**
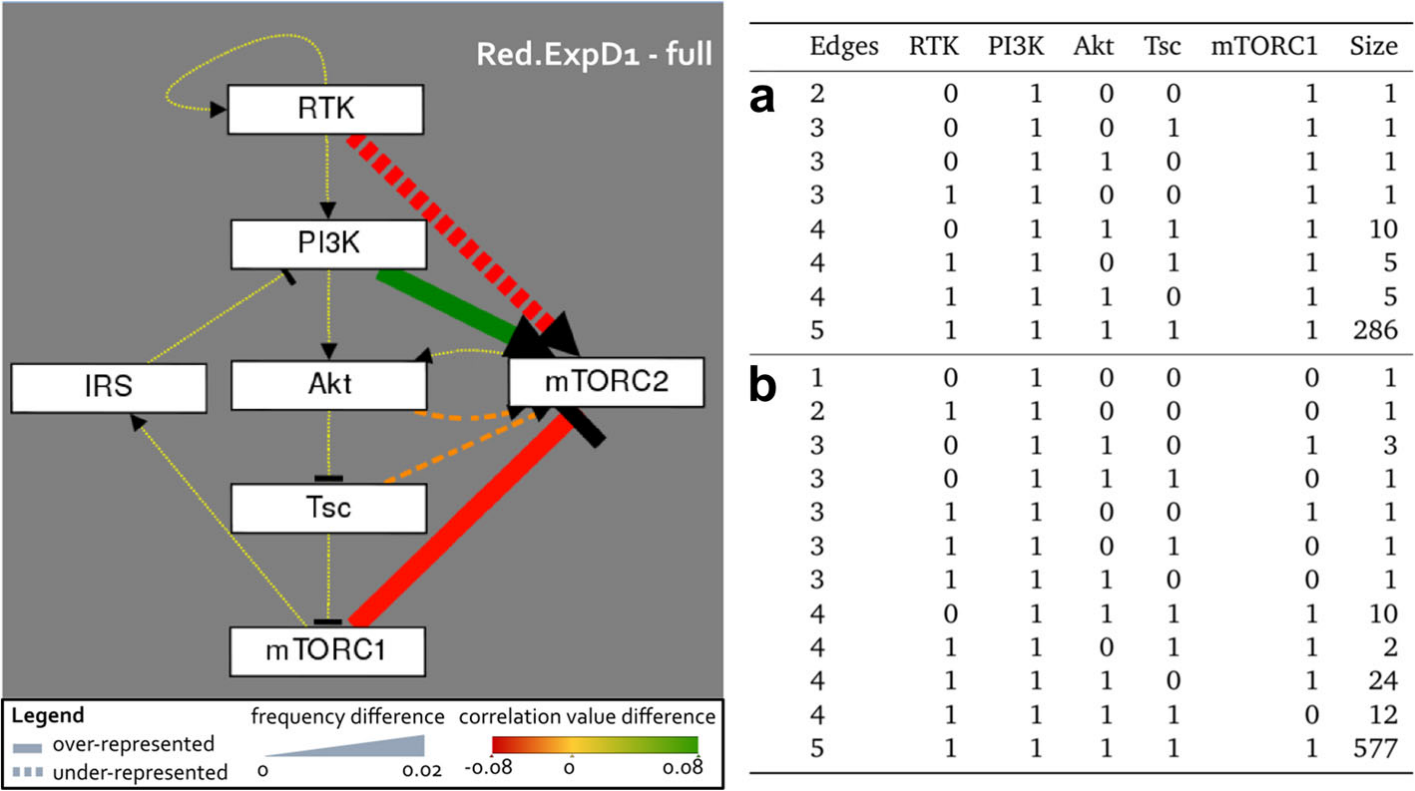
Experimental design suggests mTORC1 as second regulator of mTORC2. The model pools Red.ExpD1 and Red.ExpD2 are listed with the same classification option than Fig. 3 in Table (**a**) and (**b**), respectively. Table **a** shows the 310 models from the Red.pool that are in agreement with **ExpD1**, where all models contain PI3K and mTORC1 as essential regulators. The 634 model agreeing with **ExpD2** and Red.pool are shown in Table **b** and do not show a clear tendency towards a second regulator. The graph shows the difference of the statistical analysis of Red.ExpD1 and the initial pool, visualizing the over-representation of PI3K and mTORC1 regulation on mTORC2 and an under-representation of RTK

In order to further characterize the differences between these two pools, we analyzed both Red.ExpD1 and Red.ExpD2 analog to the Red.pool by a statistical and exact analysis. For Red.ExpD2, the results show no clear trend towards rejecting or supporting another hypothesis (Fig. 4 b). The minimal model with only the essential PI3K is in agreement with **ExpD2**, for two regulators only RTK is possible and for three regulators every hypothesis is present.

In contrast, the analysis of Red.ExpD1 identifies mTORC1 as second essential regulator, which is illustrated in the graph in Fig. 4. Here, the difference between Red.ExpD1 and the initial pool shows an increase in frequency and impact for PI3K regulation, but it also displays a over-representation of mTORC1 inhibition of mTORC2. Furthermore, RTK is under-represented in the difference graph and has a negative impact (Fig. 4). In Red.ExpD1, the minimal model contains PI3K and mTORC1 as dual regulators for mTORC2, for three regulators every other hypothesis is possible (Fig. 4 a). Thus, the data set **ExpD1** identifies a dual regulation of mTORC2 by PI3K and mTORC1 proposing an experiment to clarify this point.

## Discussion and conclusion

In this study, we used a logical modeling approach to investigate the uncertain regulation of mTORC2 by PI3K signaling. We were able to show that PI3K itself is necessary for mTORC2 activation, but the regulation is likely to be more complex. By enumerating all possible models arising from the state of the art literature, we systematically tested this pool of models for published data and analyzed the valid subpools. For analyzing these subpools, we first compared the reduced pools to the initial pool statistically. We were able to find enriched and under-represented hypotheses, but with a seemingly rather low significance.

The explanation for this issue is given by the exact analysis, where we observed that there is a bias towards models with many edges, i.e. more than 90% of models in the pool have 5 edges. There are two reasons for this bias: combinatorics and overfitting. When building the model pool, every possible logical expression is generated, where the number of combinations increases with the number of optional incoming edges. For a component with 2 regulators, the upper bound of possible truth tables is $2^{2^{2}}=2^{4}$ and for 5 regulators it is $2^{2^{5}}=2^{36}$. Also, the more regulators are allowed in a model the easier it is to produce complex dynamics, which is a common problem of overfitting.

More than 90% of the models of the initial and the specific pools have 5 edges, thus in the statistical analysis the difference for the frequency is only influenced by a maximum of 10% of the models. The impact also is biased by these models, since the impact automatically is split upon all regulators leading to a low impact in models with 5 edges. However, having 4 or even 5 kinases regulating one protein is unlikely. So even low values must be considered to uncover important trends. These are then validated in the second exact analysis to explicitly look at the minimal models of the pools.

In the exact analysis, models are grouped according to their number of optional edges. The analysis revealed that none of the hypotheses can be rejected, but require multiple edges to explain all data (see Fig. 3). On one hand this fact is surprising, because the original studies claimed different hypotheses for the mTORC2 regulation. On the other hand, the models are very similar and the data we used for filtering the pool coincided with experiments from other studies after discretizing, thus discriminating between the models is hard.

**Issue of selecting experimental data from studies** We only used a subset of the performed experiments, which are not necessarily the most weighty arguments in the studies. Here, we examined basic qualitative effects which cannot capture observations from experiments including specific manipulations of single components, such as mutating a phosphorylation site, or dose-dependent effects. For example, most data of Gan et al. contain an Akt mutant bound to the membrane which cannot be represented in our system without changing the model. Moreover, the level of detail varies among the studies, such that we selected a level of abstraction shared by all studies, e.g. we cannot include experiments with mutated SIN1, since we cannot represent a partial knock-down of mTORC2.

The experimental setups between the studies differ, from cell types to treatments and methods. Most experiments used insulin as stimulus whereas the other studies used EGF. A very interesting data set from Liu et al. (2013) showed a transient deactivation of mTORC2 for EGF on a small time-scale (0 to 60 mins) (Fig. 3d in [17]), but for insulin this effect was only observable on a large-time scale (> 60 mins) (Fig. 3b in [17]). For such long time-scales it is questionable whether the observed effect is caused by signaling processes or might involve other processes outside the model boundaries. Also, EGF stimulation mainly activates the MAPK cascade, which is known to have crosstalk effects on PI3K signaling [30]. In order to maintain a minimum level of comparability we do not use data with EGF stimulus. Nevertheless, the redundancy in the qualitative behavior in the experiments we observed in Table 1 affirms the comparability of the selected data sets.

Another data set we found to be interesting is from the study of Huang et al., which claimed to show a PI3K-independent effect on mTORC2 [16]. Here, the setup and the measured components fitted our model, so we build two different CTL formulas, **M_3BC** and **M_3BC2**, for two observations. However, the issue with this data set was that it is a single measurement after 15 minutes of stimulation, thus it is not a steady state measurement. In general, many modeling formalisms require a steady state assumption. Although we are able to test both transient states and fixpoints, testing the reachability of one transient state is easy to fulfill by the models, thus every model was valid for the transient version. For this reason, we also tested both data sets also as hypothetical fixpoints of the system.

**Comparison to original studies** The analysis in Fig. 3 revealed that PI3K is an essential regulator across all models in the filtered pools, which matches the results of Hypothesis 2 by Gan et al. even though we did not use any data from that study directly. Also, this finding supports a recent study from Yang et al. [15], where they suggested PIP_3 to act as a scaffold protein for the interaction between mTORC2 and Akt.

Comparing our results with the paper of Dalle Pezze et al., we sought for qualitative similarities and differences between the studies, since their investigations partially overlap with our studies. Since we included their final model as Hypothesis 1, we can say that our results do not rule out the existence of Hypothesis 1. In particular, the data sets from Dalle Pezze et al. **T_4B**, **T_7A** and **T_8A** do not exclude Hypothesis 1, thus there is no direct contradiction between the studies. Also their data sets have a large overlap with observations from other papers (see Table 1). However, we only used a subset of their data, since they measured the activity of mTORC2 by the phosphorylation of mTOR at S2481, for which there is a discussion on whether it is a unique read-out for the activity of the complex [31–34]. Also, for fitting the models to the data, Dalle Pezze et al. added an unknown kinase, which is assumed to also phosphorylate Akt at S473 and thereby could substitute mTORC2.

Another interesting aspect is that Dalle Pezze et al. identified a PI3K variant as mTORC2 regulator, because it is sensitive to Wortmannin, but cannot be PI3K itself due to insensitivity to the negative feedback [13, 35]. This insensitivity was observed in a knock-down experiment for Raptor, where the phosphorylation of mTORC2 did not decrease upon feedback disruption. However, Raptor knock-down deactivates mTORC1, thus it also disrupts a potential inhibition by S6K on mTORC2. Therefore, our results propose an alternative solution for the PI3K variant by having PI3K and another second regulator. Consequently, it would be very interesting to build an ODE model to be able to directly compare our models to Dalle Pezze et al. and to include quantitative information into the study.

Finally, the study of Huang et al. tested the behavior of mTORC2 in *Tsc*2^−/−^ Mef cells, where we selected three data sets. **M_1A** was the basic observation showing an impaired mTORC2 activity for the knock out, which was in agreement with almost all models in the initial pool (see Table 3). The data sets, **M_3BC** and **M_3BC2**, were not included in the Red.pool, but tested separately. The first formula, **M_3BC**, was in agreement with Red.pool showing that there is no more additional information contained in that data. The second formula, **M_3BC2**, which was the main observation of Huang et al., resulted in no intersection with the Red.pool since it directly conflicts with the formula **T_7A**.

In the experiment, they tested whether an inhibition of mTORC1 can recover the activity of PI3K on mTORC2, since it deactivates the negative feedback. Huang et al. argue that this recovery was not observed, thus they conclude that PI3K cannot solely regulate mTORC2. For this purpose, a lot of perturbations were done, Tsc knock out, Raptor knock down, stimulation, but all these actions directly affect a possible regulator of mTORC2, therefore the expressiveness of the data is limited. To address this issue, we wanted to find an experiment that does not manipulate any of the hypothesized regulators of mTORC2, but gives more information about the feedback independent processes.

**Identification of regulatory mechanisms requires deactivation of feedback** There are two major reasons, why the exact regulation of mTORC2 by the PI3K pathway is hard to identify: (i) the candidates are within one signaling cascade and (ii) the negative feedback from mTORC1 on PI3K. From the first fact the problem of very short time windows arises, where a kinase becomes active without activating its downstream target, which is also a kinase. Producing data that is able to dissect the activity of kinases in a chain reaction is hard. A possible solution is to block the cascade at different levels using inhibitors as shown in Table 1, but due to the negative feedback this treatment affects all components in the pathway.

For this reason, we proposed an experiment, where the target phosphorylation sites of the negative feedback on IRS are mutated. In detail, S302, S307 and S632 are causing a reduced signaling through PI3K when phosphorylated [36], therefore these serine residues would need to be substituted to e.g. alanine. When stimulating these mutants with insulin, we predicted two different outcomes for mTORC2, which splits the model pool in two groups for active and inactive mTORC2 in steady state. Analyzing the resulting model pools that agree with the data from the first analysis in the Red.pool, we found no clear pattern for Red.ExpD2 having active mTORC2 (Fig. 4 b). In contrast, Red.ExpD2 shows mTORC1 as a second essential regulator.

The edge from mTORC1 was reported by Liu et al. in form of a dual phosphorylation at Thr 86 and Thr 398 of the mTORC2 component SIN1. Whether or not these phosphorylations lead to mTORC2 inhibition [17] or for Thr 86 to activation by Akt as claimed by Humphrey et al. [19] is unclear, due to conflict of data [37]. Since Akt also regulates mTORC1, it remains to be clarified whether this effect is direct or indirect. Also, further studies on the exact mechanism through which modifications of SIN1 affect the mTORC2 activity are necessary [37]. Still, the modifications of SIN1 suggest that a regulation by PI3K alone might not be realistic.

Moreover, in a recent summary Yuan et al. [38] propose a dual regulation of mTORC2, with PIP_3 as scaffold and recruiter as well as S6K as inhibitor, which matches our result from the experimental design pool Red.ExpD2. This finding is also supported by a recent study in C2C12 myoblasts using ODE modeling, where a regulation by PIP3 and S6K is proposed [39].

These considerations show that a potentially interesting next step could be to construct more detailed models in terms of modeling formalism or resolution of components, like mTORC2, to increase comparability and more fully exploit the available data. Such a study could lift our qualitative results to a more quantitative understanding of the mechanism of regulation of mTORC2.

## Methods

In this paper, an approach for systematically analyzing uncertain biological systems by logical modeling is used. For this aim, background on workflow, formalism and tools are given as well as information about data processing.

### Modeling uncertain systems

**Fig. 5:**
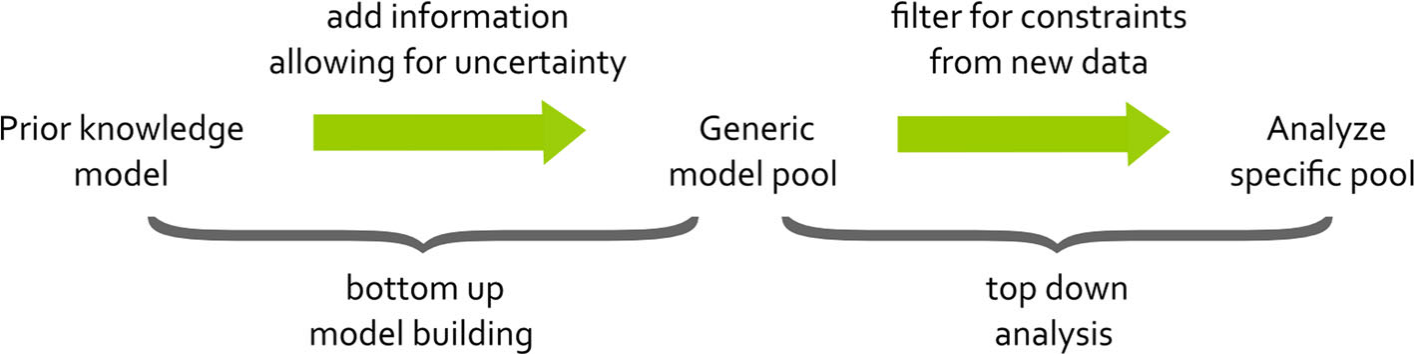
Workflow for modeling approach. First a generic model pool is created from all available information including uncertainty. Then the pool is filtered for data to find specific subpools, which can be analyzed for new properties

In the approach, bottom-up model building is used to collect all available information about the system in a generic model pool, which is subsequently reduced by testing consistency with data and analyzed for properties (see Fig. 5). In detail, all known and uncertain information about interactions and regulatory mechanisms is collected. Then, the topology of the model is represented as a graph with known edges labeled as mandatory and uncertain edges as optional as well as the nature of the interaction being positive or negative. The combinations of all optional edges and all possible regulatory mechanisms between them give rise to the generic model pool.

The generic model pool might contain models that are not consistent with data from literature studies. Therefore, this data is formalized and compared with the dynamical behavior of every model in the pool. This process can be computationally expensive, since the number of models can quickly add up to thousands of models. For this reason, a tailored model checking software for efficient analysis is employed, which filters and evaluates the specific pool, i.e. Tremppi [21] and TomClass [20].

**Background on logical modeling** In this paper, Boolean networks are used, where components can adopt the value 0 for inactive and 1 for active state. The topology of a regulatory system is described as a directed graph *R*={*V,E,l*}, where the components *V*={1,…,*n*} are represented as nodes, the interactions *E*⊆*VxV* are represented as edges and marked with edge labels *l*:*E*→*L* adapted to the definition from [20]. *L* is a set of formulas with + for activating and − for inhibiting edge sign, describing the effect of the regulator through that edge on its target. Here, we assign the label + *or* − to edges of known interaction from literature that were always observable with an activating or inhibiting effect, respectively. The label ¬+ (¬−) is assigned to uncertain edges meaning there were contradicting studies in literature showing inhibiting (activating), but in other studies no effect.

Then for each component a function $f: \mathbb {B}^{n} \to \mathbb {B}=\{0,1\}$ is defined that specifies the impact of all regulators on its target according to the edges labels (more details see [20]). In case there is more than one regulator controlling a component, logical operators define the connection between them, i.e. ∧ for logical AND connection, ∨ for logical OR and ¬ for logical negation. Finally, a model contains one unique function for every component. Since some components have uncertainty in their edge labels and regulatory mechanisms, there exists more than one possible function. Thus, every combination of functions across all components give rise to the model pool.

A system state $X= (x_{v}) \in \mathbb {B}^{n}$ describes the value of each component *v*∈*V* of a model. Then, the dynamical behavior is derived from by the functions *f* using an update to define the transitions between states. Here, we employ an asynchronous update to the functions [40], where only one component is changed at a time, which usually results in non-deterministic behavior but includes good matches for biological behavior.

**Discretization and formal encoding of data** In order to apply constraints from data to reduce and validate a model pool, the data is discretized to match the logical formalism. The process of discretization is a strong simplification that comes with a loss of information especially for quantitative data, but can reduce the effects of noise in data. However, the qualitative effects, such as activation or inhibition of a component, as well as high-level behavior, such as stability, are preserved. Therefore, the data is processed in two steps: (i) discretization of the data and assignment to the designated readout component, and (ii) formalization of temporal observation such as time series data.

(i) For discretizing data points into two states, a threshold needs to be defined. In case there is quantitative information available, different methods can be applied to find a threshold, such as mean or median [41]. For qualitative data, measurements are usually interpreted relative to a control measurement. If a measurement is ambiguous, it is excluded from the study. Since the activity of a protein kinase can be measured by the phosphorylation of its target, we define the presence of target phosphorylation as readout of the activity of a component.

(ii) In this study, two different kinds of data sets are included: time series measurements and single measurements. All measurements need to be interpreted dynamically and encoded so they can be utilized in automated verification. Here, we use temporal logics CTL for formalizing the data. For time series experiments, each measurement represents a transient state of the system, where the first measurement is called initial state and the following measurements are represented as a sequence in the order of time. Each model is then tested for consistency with the data by checking whether it can generate a path containing the sequence. If measurements do not specify a unique state, i.e., if data for some components is missing, all states in agreement with the measured values are considered. Note that any kind of change between two states in the sequence is allowed as long as the system manages to reach the next state at some point. Single measurements are often assumed to be steady states of the system after e.g. perturbations like knock-outs. A steady state is described as a stable long term behavior and can be compared with fixpoints of logical models. For denoting the CTL formulas, the following semantics are used:

- EF(X): is a CTL operator *exists finally*. This states that on some path from an initial state the X holds true at some point.
- Delta=0: states that no change is possible, i.e. we are in a steady state.
- v=b: where $\texttt {v} \in V, \texttt {b} \in \mathbb {B}$ states that value of a component v is set to b.
- Initial state: is a list of boolean constraints on the values of the components. A state is considered initial, if all the constraints are satisfied.
- Fixed component: constrains the listed components to the assigned values for the whole path. This property allows for modeling knock-outs and stimuli.
